# Correspondence

**Published:** 1887-06

**Authors:** 


### CORRESPONDENCE.

Dr. E. J. Lee, Philadelphia.

Dear Sir:—I have been looking for a good homœopathic monthly, and the other day came across a copy of The Homœopathic Physician, which seemed to meet my desires excellently, and I have decided to become a subscriber to it. The copy was three years old, and fearing that there might have been a removal or change of some kind, I thought it best to write your old address before sending money, and ascertain whether the Physician is holding forth at the old stand.

I am not a physician, but a lay brother strong in the faith, and a high potency man. I have made a study of medicine, commencing six years ago to cure myself of a nervous disorder.

After having passed through the hands of the Dean and others of the faculty of the Cleveland Homœopathic College, and two local homœopaths, I found myself getting *no better* very fast. Instead of blaming the practice, I charged the matter to the physicians and set about looking up my own complaint. Well, to make a long story short, I succeeded in curing myself radically, and there is no heartier man than I am to-day, I believe. My researches led to the discovery that not one of fifty so-called homœopathic physicians practices Homœopathy, and very few are fitted to practice it. Another thing, none of the so-called homœopathic periodicals that I have examined (except the PHYSICIAN), *are* homœopathic. Probably this is neither news nor interesting to you, but I like to relieve my mind once in a while. I have a good homœopathic library and doctor my own family. Took three of my children through good cases of diphtheria this winter, one of whom, a four-year-old, had the membrane in nostrils. Will send money for subscription when I hear from you.

Respectfully, E. S. W.

